# Global Economic Burden of Spinal Muscular Atrophy: A Systematic Literature Review

**DOI:** 10.7759/cureus.81023

**Published:** 2025-03-23

**Authors:** Rawda Elshahawy, Baher Elezbawy, Rasha Ashmawy, Rowan Elshahawy, Yosra S Mahmoud, Nada Korra, Sherif Abaza, Amal Alnajjar, Hana A Al-Abdulkarim, Hussain A Al-Omar, Sahar Fahmy, Sara Al Dallal, Ahmad N Fasseeh

**Affiliations:** 1 Health Economics, Syreon Middle East, Alexandria, EGY; 2 Evidence Synthesis, Syreon Middle East, Alexandria, EGY; 3 Health Technology Assessment, Semmelweis University Doctoral School, Budapest, HUN; 4 Clinical Research, Maamora Chest Hospital, Alexandria, EGY; 5 Statistics, Syreon Middle East, Alexandria, EGY; 6 Health Economics, Syreon Middle East, Cairo, EGY; 7 Drug and Poison Information Services, Security Forces Hospital, Riyadh, SAU; 8 King Abdullah International Medical Research Center, National Guard Health Affairs, Riyadh, SAU; 9 Doctoral School Applied Informatics and Applied Mathematics, Óbuda University, Budapest, HUN; 10 Clinical Pharmacy, College of Pharmacy, King Saud University, Riyadh, SAU; 11 Undersecretary Office, Department of Health, Abu Dhabi, ARE; 12 Health Service, Dubai Health Authority, Dubai, ARE; 13 Emirates Health Economics Society, Emirates Medical Association, Dubai, ARE; 14 Modelling, Syreon Middle East, Alexandria, EGY; 15 Pharmacy, Alexandria University, Alexandria, EGY

**Keywords:** cost, disease burden, economic burden, productivity loss, resource utilization, sma, spinal muscular atrophy

## Abstract

Spinal muscular atrophy (SMA) is a rare inherited neuromuscular disease classified into four main subtypes and characterized by severe muscle weakness and loss of motor function. Its high mortality rates, high treatment costs, and lengthy care requirements place a heavy burden on patients, caregivers, and the healthcare system. This study aims to explore the economic burden of SMA subtypes by analyzing costs, healthcare resource use, and loss of productivity for patients and their caregivers.

We conducted a systematic literature review, searching for studies published since 2010 via Medline, Embase, Google Scholar, and gray literature databases. We extracted data concerning costs, healthcare resources, and productivity losses among SMA subtypes. The quality of the included studies was assessed using the Newcastle-Ottawa Scale and the Quality of Health Economic Studies tools.

We retrieved 55 studies from 32 countries with economic data variation due to the study design, location, and SMA subtype. The weighted average annual cost for an SMA patient was US$109,906 with the highest costs observed in type 1 patients, who incurred direct medical costs without disease-modifying treatments of US$187,88. The non-medical costs accounted for US$109,379 per patient, along with frequent hospitalizations and high caregiver productivity losses, requiring 2,947 hours of caregiving annually.

The direct and indirect costs of SMA are substantial. The necessity for standardized approaches to evaluate and analyze the economic impact across various SMA subtypes is highlighted by the heterogeneity of the data. In order to control the financial burden of SMA, governments and healthcare systems can benefit from these insights to develop policies aimed at improving financial sustainability and patient support.

## Introduction and background

Spinal muscular atrophy (SMA) is a group of genetic neuromuscular disorders that involves the loss of muscle nerve cells, resulting in muscle weakness, atrophy, low muscle tone, and impaired movement. The disease originates from the loss or mutations of the survival motor neuron (SMN) gene, leading to reduced SMN protein levels, which is responsible for the functionality of motor neurons. SMA manifests in proximal muscles such as the shoulders, hips, and back. It also causes impairment of vital functions such as feeding, swallowing, and breathing and affects most organs controlled by voluntary muscles in the patient’s body [1,2]. Since it only affects a limited proportion of the population, SMA is considered a rare disease [3]. Studies report incidence values ranging from one in 6,000 to one in 10,000 live births and a prevalence of approximately 1-2 per 100,000 persons [4,5]. However, the disease still imposes a huge burden on healthcare systems and society due to its high mortality rates and economic burden, especially in its severe forms [6].

There are four main types of SMA that have been classified: from SMA type 1 to type 4 according to the age at onset and the maximum motor milestone achieved. SMA type 1 is considered the most severe type, while SMA type 4 is the least in severity with rare prevalence [7]. Additionally, there is another subtype, known as SMA type 0 or prenatal SMA, which is considered a fifth type. Symptoms of SMA type 0 appear before birth. However, this type is difficult to diagnose, as affected infants may die at birth or a few months after [8]. Globally, patients with SMA types 1, 2, and 3 represent around 99% of the SMA patient population; therefore, most studies focus on these subtypes, while type 0 and type 4 are less prevalent [9,10]. Managing such a severe disease involves costly interventions and healthcare resources to manage respiratory problems and swallowing difficulties, among several other symptoms [11]. Additionally, the novel treatments approved for SMA are expensive, further exacerbating the economic burden. These therapies, while offering significant clinical benefits, require substantial financial investment from both healthcare systems and families [12].

Another factor that contributes to the disease’s economic burden is that most SMA patients are infants or children, due to the age-onset nature of the disease, except for type 4. This implies the requirement of extensive care, so the disease burden does not stop at the patient level but extends to their formal and informal caregivers, creating a substantial impact on their productivity. Even adults with SMA are usually unable to perform their daily activities independently and rely on caregivers’ support to perform their activities [13]. The economic burden of SMA encompasses direct medical costs (e.g., treatment, hospitalizations, and medications), direct non-medical costs (e.g., assistive devices, transportation, and home modifications), and indirect costs (e.g., productivity losses for both patients and caregivers), all of which vary among SMA subtypes. Several recent systematic literature reviews (SLRs) have explored the economic burden of SMA; however, none have provided a comprehensive quantification of all detailed components of the economic burden or calculated the weighted average values for these components within the same study [14-20]. While burden-of-disease studies aim to support decision-makers in making informed choices, the absence of a complete, quantified burden limits their applicability to inform specific policy or healthcare decisions, rendering them more general in scope [21].

Moreover, the existing literature is fragmented, particularly in terms of inconsistent categorizations and taxonomies used to report costs and productivity losses, making cross-study comparisons and aggregated analyses challenging [16]. Data on productivity losses associated with SMA, including key factors like absenteeism, presenteeism, and workplace accommodations, are limited and inconsistently reported. These gaps underscore the need for a systematic, comprehensive analysis of costs, healthcare resource utilization (HCRU), and productivity loss across SMA subtypes. To address these issues, this study aimed to provide detailed estimates for all cost components, including direct medical costs, indirect costs (such as productivity losses), and non-medical costs, associated with SMA and its subtypes. By employing a standardized methodology, our SLR synthesizes and analyzes economic data to ensure comparability and reliability, while providing decision-makers with actionable insights.

## Review

We conducted an SLR and reported its results in accordance with the Preferred Reporting Items for Systematic Reviews and Meta-Analyses (PRISMA) 2020 guidelines [22]. We searched for studies that provided numeric values for at least one of the study domains, namely, cost, HCRU, or productivity loss for SMA patients and caregivers.

Search strategy

A comprehensive search was conducted on March 17, 2022, using the electronic databases Medline (via PubMed search engine) and Embase (via Scopus search engine). The search term consisted of different combinations of the following domains and their synonyms: “Spinal muscular atrophy”, “cost”, “health care resource utilization”, and “productivity lost”. We also searched gray literature sources to make sure we did not miss any potentially relevant studies; Google Scholar was searched using specific domains, and we screened the first 100 hits. The search term domains, detailed search terms, and search strategies are presented in Supplemental materials 1 and 2.

The search process was limited to studies published from 2010; any older studies were excluded to focus on the most recent evidence and because costs and resource utilization data change frequently, based on technological advancement and lifestyle changes. Also, several treatments have been developed during this period, which contribute to the economic impact and prognosis of the disease [23,24]. We also restricted our search to studies published in the English language. No geographical restriction was applied.

Study selection process

Title and Abstract Screening

Title and abstract screening was conducted via Rayyan online software (Rayyan Systems Inc., Cambridge, MA, US). Duplicates were resolved through an automatic software feature [25]; then, the following exclusion criteria were applied in hierarchical order: 1) publications with no English abstract; 2) studies published before January 1, 2010; 3) studies that are irrelevant to SMA; 4) irrelevant study design (animal studies, in vitro studies, editorials, letters, non-systematic reviews, and case reports); 5) studies including patients with SMA and another confounding disease; 6) studies that do not include economic burden data (no cost, resource utilization, or productivity loss data); 7) and SMA type 0. Relevant systematic reviews were processed separately, with their references checked to ensure no relevant studies were missed. Each study was screened by two independent reviewers, and any disagreement was resolved by a third one.

Full-Text Screening

Full-text screening applied the same exclusion criteria as the title and abstract phase, with the addition of excluding inaccessible studies. Each study was screened by two independent reviewers, and any disagreement was resolved by a third one.

Data extraction

Reviewers carried out a pilot extraction; then, the data extraction sheet was adjusted and finalized according to their suggestions. Relevant data was extracted in Microsoft Excel® (Microsoft Corp., Redmond, WA, US).

The following data were extracted from eligible studies: patients' age; number of patients; type of cost reported; cost value; perspective; currency; HCRU type, value, and measurement unit; and productivity loss type, value, and measurement unit. The detailed data extraction sheet domains are shown in Supplemental material 3. For each study, one reviewer extracted the relevant data, and another validated the extraction for accuracy and completeness. Any disagreement between the reviewers was resolved by discussion between them.

Data analysis

The extracted data were analyzed and compared using appropriate statistical and economic evaluation tools. We conducted quantitative data synthesis and generated descriptive statistics (frequencies and percentages) for study characteristics, including study countries, average patient age, and SMA subtypes. Additionally, we calculated weighted average costs and performed economic evaluations to compare SMA subtypes across various cost components (total cost, total direct cost, direct medical cost, direct non-medical cost, and indirect cost), healthcare utilization metrics (diagnostic utilization, hospitalizations, outpatient visits, respiratory support, and rehabilitation center visits), and productivity loss for both caregivers and patients.

Costs

**Figure 1:**
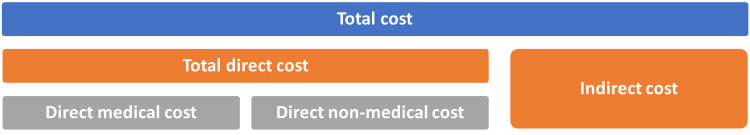
Cost categorization used for cost data extraction The figure illustrates the breakdown of total cost (blue) into total direct cost (orange) and indirect cost (orange). Total direct cost is further divided into direct medical costs (gray), which include healthcare-related expenses like hospital stays and medications, and direct non-medical costs (gray), covering expenses such as transportation and caregiving. Indirect cost represents productivity losses due to illness or disability. Figure created by the authors.

Different types of costs were reported in the included studies, and each referred to cost items in different taxonomies or categorizations. To be able to create average values from several studies, we defined specific categorizations, and when extracting data from the studies, we fitted the extracted data into these categories, to make sure no different data would be grouped together because they used different taxonomy (e.g., some studies referred to total cost as the sum of direct and indirect costs, while others referred to total cost as the sum of direct medical costs only; here, we did not take the average of both values as the total cost, but we grouped them according to our categorization). We grouped costs into five categories: total cost, total direct cost, direct medical cost, indirect medical cost, and indirect cost. The categorization is illustrated in Figure 1.

Total costs were defined as all relevant costs (direct costs + indirect costs), while total direct costs were divided into direct medical costs (hospitalization, medications, and diagnostics costs) and direct non-medical costs (house and vehicle modification costs, transportation, and travel expenses). Indirect costs involve productivity loss by patients and their caregivers.

All costs extracted were converted to annual cost per patient to include in the aggregated analysis. If a study reported its time horizon as “lifetime,” we searched for the average life expectancy in the study to estimate the relevant period. If the number of patients was not mentioned-especially in economic evaluation studies-we assumed the number of patients to be 10 according to economic evaluations' good practices [26].

Costs were reported in different currencies and at different timepoints by the included studies. We adjusted the cost values to inflation using the consumer price index for 2020 from the World Bank database [27], and then, values were converted to US dollars of 2020 using the official exchange rate from the World Bank database [28].

For cost analysis, we categorized SMA patients into type 1 only; type 2 only; type 3 only; types 1, 2, and 3; and unspecified SMA types. We excluded data reporting detailed cost subcategories from the analysis like subcomponents of the direct medical costs (ex: cost of specialist visits and cost of hospitalization) to prevent double counting. Instead, we analyzed data reported as a total direct cost or total direct medical cost.

Healthcare Resource Utilization

The pilot data extraction phase helped to identify the most common resources utilized by SMA patients. Data were extracted for all available subtypes showing the average use of each resource by SMA patients. Similar resources were aggregated, and then, a weighted average for each resource was calculated based on the number of patients for each reported value. Values were differentiated by the SMA subtype.

There were several different resource utilization items reported in each study. The resources that had at least three data points reported among all included studies were included in the analysis to guarantee an accepted level of reliability. The extracted data was further checked for eligibility to include in the aggregated analysis. Studies that did not report a specific time horizon were excluded from further analysis, due to the inability to calculate the annual cost per patient.

Productivity Loss

We extracted data from all studies that reported productivity loss for patients or caregivers due to SMA. Absenteeism was defined as the number of days that a patient is absent from work or school, while presenteeism was defined as the number of days the patient is at work or school but is unproductive [29].

Productivity loss was reported either as the number of days or hours lost during a certain period or as the productivity percentage lost. All productivity loss values were adjusted to the number of hours lost annually per patient or caregiver. Due to the scarcity of data on each type of productivity (absenteeism or presenteeism), the aggregated data was represented as time lost in general. For the analysis, we included type 1 only; type 2 only; type 3 only; types 1, 2, and 3; and unspecified SMA types. We excluded data points with no clear information about the time lost (e.g., percentage of patients who need from eight to 16 hours of care).

Quality assessment

We assessed the quality of the included studies using different tools based on the study design. The Newcastle-Ottawa Scale (NOS) for cohort studies evaluated the selection, comparability, and outcome domains of the included studies, providing scores categorized as poor, fair, or good quality. The NOS tool for cross-sectional studies provided scores based on quality categorized as very good, good, satisfactory, or unsatisfactory [30,31]. For economic evaluations, we used the Quality of Health Economic Studies (QHES) tool, which assesses 16 items related to methodology, data transparency, and analysis robustness, with scores ranging from 0 to 100 (higher scores indicating better quality) [32]. Each study was independently evaluated by two reviewers, with discrepancies resolved through discussion or a third reviewer.

Study selection and summary of included studies

**Figure 2:**
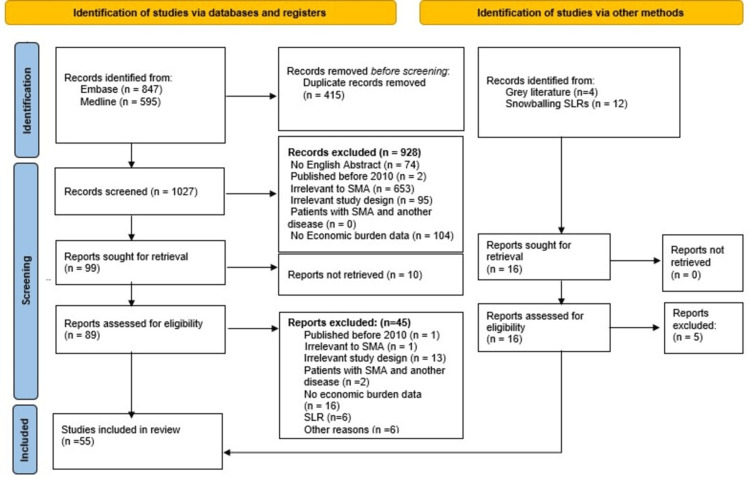
PRISMA flow chart of the study selection process of the review PRISMA flow diagram created by the authors based on the PRISMA 2020 guidelines (Page et al. [22]). SLR: systematic literature review; SMA: spinal muscular atrophy; PRISMA: Preferred Reporting Items for Systematic Reviews and Meta-Analyses

Initially, 1,442 records were identified from databases with an additional 16 records from gray literature and snowballing references from systematic reviews. After removing 415 duplicates, 1,027 records underwent screening, where 928 were excluded due to the above-mentioned exclusion criteria. Following this, 99 studies were sought for retrieval, but 10 were not retrieved, leaving 89 studies assessed for eligibility. Out of these, 45 studies were excluded, leading to the inclusion of 55 studies in the final review. Figure 2 shows the PRISMA flow diagram of the study selection process.

Table 1 summarizes the studies included in the analysis, highlighting key characteristics such as study period, sample size, SMA subtype, patient age, and cost, productivity loss, and HCRU. Of these, 34 studies reported cost-related data, 39 included HCRU, and 10 addressed productivity loss. The mean and median ages of patients were 14.34 and 7.86 years, respectively, with type 1 SMA patients averaging 1.52 years of age and type 2 averaging 6.55 years. Most studies commonly reported SMA subtype 1 (31%), followed by those including subtypes 1, 2, and 3 together or unspecified SMA subtypes. Studies on subtype 2 alone were also frequent, while other combinations were less common. The distribution of SMA subtypes across studies is illustrated in Supplemental material 4.

**Table 1:** Summary of included studies The table shows all included studies in the systematic review and presents what each of these studies includes regarding costs, productivity loss, or healthcare resource utilization (HCRU) data. N.D.: not defined; SMA: spinal muscular atrophy

#	Author (year)	Study period in years	No. of patients/caregivers	Name of the group	Type of SMA	Age (years)	Includes cost data	Includes productivity loss data	Includes HCRU data
1	Ali et al., 2019 [33]	3 (2017-2019)	11	SMA patients	Unspecified	N.D.	Yes	No	Yes
2	Al-Zaidy et al., 2019 [34]	2 (2014-2015)	12	SMA patients	1 only	Mean (0.28)	No	No	Yes
3	Arjunji et al., 2019 [35]	N.D.	92	SMA patients	1 only	N.D.	No	No	Yes
4	Armstrong et al., 2016 [36]	N.D.	239	SMA patients	Unspecified	N.D.	Yes	No	Yes
5	Barbour et al., 2021 [37]	6 (2015-2020)	3,775	SMA patients	1, 2, 3, & 4	Median (13.41)	No	No	Yes
6	López-Bastida et al., 2017 [38]	1 (2015)	81	SMA patients	1, 2, & 3	Mean (7.22)	Yes	Yes	No
7	Lopez Bastida et al., 2019 [39]	1 (2015)	86	SMA patients	1, 2, & 3	N.D.	Yes	No	No
8	Beauchamp et al., 2019 [40]	N.D.	6	SMA patients	Unspecified	N.D.	Yes	No	No
9	Belter et al., 2020 [41]	5 (2012-2016)	393	All SMA patients	Unspecified	N.D.	Yes	No	No
10	Bielsky et al., 2018 [42]	3 (2015-2017)	8	SMA patients	2 only	Median (4.1)	No	No	Yes
11	Bladen et al., 2014 [43]	N.D.	5,068	SMA patients	1, 2, & 3	N.D.	No	No	Yes
12	Broekhoff et al., 2021 [44]	N.D.	N.D.	SMA patients	1 only	Mean (2.83)	Yes	No	No
13	CADTH, 2019 [45]	N.D.	N.D.	SMA patients	1, 2, & 3	N.D.	Yes	No	No
14	Cardenas et al., 2019 [46]	N.D.	237	SMA patients	1 only	N.D.	Yes	No	Yes
15	Chambers et al., 2020 [47]	2 (2016-2017)	40	SMA patients	1, 2, & 3	Average (9.38)	Yes	No	Yes
16	Chen et al., 2021 A [48]	4 (2016-2019)	9	SMA patients	1 only	Median (0.88)	No	No	Yes
17	Chen et al., 2021 B [49]	6 (2012-2017)	49	Pediatric-onset SMA	Unspecified	Mean (5.5)	Yes	No	Yes
18	Cremers et al., 2019 [50]	6 (2010-2015)	48	Mothers of home-living patients with SMA	Unspecified	Mean (12.2)	No	No	Yes
19	Dabbous et al., 2018 [51]	6 (2011-2016)	119	SMA patients	1 only	N.D.	Yes	No	Yes
20	Darbà and Marsà, 2019 [52]	19 (1997-2015)	705	SMA patients	Unspecified	Mean (37.0)	Yes	No	Yes
21	Darbà, 2020 A [53]	4 (2014-2017)	396	SMA patients	1, 2, 3, & 4	Mean (57.3)	Yes	No	Yes
22	Darbà et al., 2020 B [54]	11 (2007-2017)	524	SMA patients	1, 2, 3, & 4	Mean (38.3)	No	No	Yes
23	Dean et al., 2021 [55]	N.D.	N.D.	Updated model	1 only	N.D.	Yes	No	No
24	Droege et al., 2020 A [56]	3 (2016-2018)	6,526	Whole cohort (SMA 1 + other SMA)	1, 2, 3, & 4	N.D.	Yes	No	Yes
25	Droege et al., 2020 B [57]	4 (2016-2019)	449	SMA type 1 patients	1 only	N.D.	No	No	Yes
26	Farrar et al., 2018 [58]	1 (2016)	8	Whole cohort	2 & 3	Mean (6.4)	No	Yes	No
27	Fox, 2020 [59]	4 (2015-2018)	704	SMA type 1 patients and SMA others	1, 2, 3, & 4	N.D.	Yes	No	Yes
28	García-Salido et al., 2015 [60]	3 (2010-2012)	9	Whole cohort	1 only	N.D.	No	No	Yes
29	Gauthier-Loiselle et al., 2021 [61]	4 (2016-2019)	324	Whole cohort	1, 2, & 3	N.D.	Yes	No	No
30	Gonçalves et al., 2021 [62]	N.D.	101	Total SMA type 1 patients	1 only	N.D.	No	No	Yes
31	Han et al., 2015 [63]	14 (2000-2013)	33	SMA patients	1 & 2	N.D.	No	No	Yes
32	Hully et al., 2020 [64]	5 (2012-2016)	80	SMA 1 patients	1 only	N.D.	No	No	Yes
33	ICER, 2019 [65]	N.D.	N.D.	Early- and late-onset SMA patients and presymptomatic SMA patients	1, 2, & 3	N.D.	Yes	Yes	Yes
34	Jalali et al., 2020 [66]	N.D.	N.D.	SMA type 1 patients	1 only	N.D.	Yes	No	No
35	Johnson et al., 2021 [67]	8 (2007-2014)	446	Total population	Unspecified	Mean (45.0)	Yes	No	Yes
36	Kao et al., 2019 [68]	11 (2005-2015)	15	SMA patients	Unspecified	Mean (8.5)	No	No	Yes
37	Klug et al., 2016 [69]	1 (2013)	189	SMA patients	1, 2, & 3	Median (19.0)	Yes	Yes	Yes
38	Lee et al., 2019 [70]	9 (2005-2013)	229	SMA patients	Unspecified	N.D.	Yes	No	Yes
39	Lemoine et al., 2012 [71]	8 (2002-2009)	49	SMA patients	1 only	N.D.	Yes	No	Yes
40	The Lewin Group, 2012 [72]	1 (2009)	N.D.	Ealy SMA and other SMA patients	1, 2, 3, & 4	N.D.	Yes	Yes	Yes
41	Lomba et al., 2021 [73]	N.D.	4	SMA patients	1 only	Mean (2.82)	No	No	Yes
42	Peña-Longobardo et al., 2020 [74]	1 (2015)	86	SMA patients	1, 2, & 3	N.D.	Yes	Yes	No
43	Malone et al., 2019 [75]	N.D.	N.D.	SMA patients	1 only	N.D.	Yes	No	Yes
44	McMillan et al., 2021 [76]	1 (2020)	1927	SMA patients and caregivers	1, 2, & 3	N.D.	Yes	Yes	Yes
45	Moran et al., 2015 [77]	N.D.	6	SMA type 2	2 only	Median (9.0)	No	No	Yes
46	NICE, 2019 [78]	N.D.	N.D.	Early- and late-onset SMA patients	1, 2, & 3	N.D.	Yes	No	No
47	Ottonello et al., 2011 [79]	5 (2006-2010)	16	SMA patients	1 only	Mean (3.38)	No	No	Yes
48	Aranda-Reneo et al., 2020 [80]	1 (2015)	68	SMA patients	1, 2, & 3	Mean (7.0)	No	Yes	No
49	Rowell et al., 2020 [81]	N.D.	122	SMA patients	1, 2, & 3	N.D.	No	Yes	Yes
50	Shih et al., 2021 [82]	N.D.	N.D.	SMA patients	1, 2, & 3	N.D.	Yes	No	No
51	Tan et al., 2019 [83]	11 (2006-2016)	341	SMA patients	1, 2, 3, & 4	N.D.	Yes	No	Yes
52	Tassie et al., 2013 [84]	2 (2010-2011)	35	SMA patients	1 only	N.D.	No	No	Yes
53	Tetafort et al., 2017 [85]	2 (2014-2015)	915	Infantile and inherited SMA patients	N.D.	N.D.	Yes	No	No
54	Thokala et al., 2020 [86]	N.D.	1	SMA patients	N.D.	N.D.	Yes	No	No
55	Zuluaga‑Sanchez et al., 2019 [87]	2 (2017-2018)	1	SMA patients	1, 2, 3, & 4	N.D.	Yes	Yes	Yes

The majority of the included studies were either cross-sectional (15 studies) or cohort (29 studies), with 11 studies being economic in nature (Supplemental material 5). According to the quality assessment, 38 studies have received ratings of "good" or "very good," with only five classified as “very good.” Six studies, however, have been assessed as "unsatisfactory," mostly because of limitations in the reporting, sample size, or design. Economic evaluations, on the other hand, show excellent quality, scoring between 86 and 100.

The relevant studies included data from 32 different countries. The United States was involved in the largest number of included studies (n, %), followed by the United Kingdom, Spain, Australia, France, and Germany. The number of times each country was involved in the included studies is shown in Supplemental material 6, provided that those countries were involved in three studies or more.

Costs

Thirty-four studies reported cost data. The sum of the weighted average for studies reporting total costs, total direct costs, direct medical costs, direct non-medical costs, and indirect costs is reported in Table 2 and Supplementary materials 7 and 8.

**Table 2:** Average annual cost for each SMA type patient (weighted) in US$ based on reported values The table shows a summary of average direct medical, direct non-medical, and indirect SMA costs reported in the included studies and shows the source of these average value calculations. n: number of studies; US$: United States dollars; SMA: spinal muscular atrophy

Cost type	Type 1 only		Type 2 only		Type 3 only	
	Cost (US$)	Studies reporting cost	Cost (US$)	Studies reporting cost	Cost (US$)	Studies reporting cost
Direct medical cost	187,881	n = 11 [44,45,51,56,59,61,69,71,78,83,87]	50,508	n = 4 [38,45,61,69]	77,942	n = 3 [45,61,69]
Direct non-medical cost	109,379	n = 2 [69,87]	52,260	n = 2 [38,69]	32,366	n = 1 [69]
Indirect cost	25,070	n = 3 [47,69,87]	19,071	n = 2 [47,69]	16,233	n = 2 [32,41]

For subtype 1 SMA, the direct medical cost weighted average is US$187,881 (from 11 studies), the direct non-medical cost is US$109,379 (from two studies), and the indirect cost is US$25,070 (from three studies). For subtype 2 SMA, the direct medical cost weighted average is US$50,508 (from four studies), the direct non-medical cost is US$52,260 (from two studies), and the indirect cost is US$19,071 (from two studies). Type 3 SMA accounted for a weighted average of US$77,942 for direct medical costs, US$32,366 for direct non-medical costs, and US$16,233 for indirect costs, based on three studies. These results demonstrate the significant cost variations among SMA subtypes, with type 1 exhibiting the greatest financial impact (Table 2).

The weighted average total annual costs for SMA patients are presented in Supplemental material 7, which demonstrates significant diversity among studies. The total reported annual costs range from US$3,752 [47] to US$593,517 [40], with an average total cost per patient of US$109,096. For direct costs, a minimum of US$2,103 [47] and a maximum of US$180,052 [87] were reported, resulting in an average direct cost per patient of $58,412. With an average of US$59,570, direct medical costs were very high and varied from U$0 [72] to US$334,715 [51], demonstrating the fluctuation of treatment costs. However, the data from The Lewin Group was based on estimates from a small sample size [72].

The economic burden on caregivers appears in the average of US$39,910 for direct non-medical costs, which range from US$16,967 [72] to US$141,893 [87]. Moreover, the indirect costs including loss of productivity averaged US$18,025, with reported values ranging from US$1,649 [47] to US$58,796 [87]. These variations highlight the significant and diverse economic burden of SMA, emphasizing the need for standardized cost assessments to guide healthcare policy and resource allocation.

For each SMA subtype, the weighted average direct medical, direct non-medical, and indirect expenses as reported by individual studies are summarized in Supplemental material 8. Type 1 SMA has the highest costs, according to the analysis, which also shows high-cost differences among subtypes. For example, direct medical costs for SMA subtypes varied significantly across studies: type 1 SMA ranged from US$235,198 to US$334,715, type 2 SMA ranged from US$43,571 to US$100,450, and type 3 SMA ranged from US$5,453 to US$43,327, with the lowest expenditures reported for type 3 SMA. These ranges are based on reported results from relevant studies [38,47,51,59,61,69].

Healthcare resource utilization

The HCRU by each SMA subtype is compared more thoroughly in Table 3. In terms of emergency room (ER) visits, patients with type 1 SMA have the greatest rate, averaging 1.27 ER visits/year, accounting for 78.45% of the patient population (n = 5 studies). On the other hand, type 2 and 3 SMAs show owner percentages of ER admissions (51.2% and 36.9%, respectively, from a single study), compared to type 1.

**Table 3:** Average annual healthcare resource utilization (HCRU) per SMA type The table presents the reported HCRU cost components by SMA subtype and shows the reported average values and the sources used for these average value calculations. LOS: length of stay; n: number of studies; SMA: spinal muscular atrophy

HCRU	Type 1 only	Type 2 only	Type 3 only	Type 4 only	Unspecified	Types 1, 2, & 3	Types 1, 2, 3, & 4
Emergency room visits (number of admissions per year, % of patients)	1.3 admissions, 78.5%, n = 5 [33,48,71,76,83]	51.2%, n = 1 [76]	36.9%, n = 1 [76]	-	0.3 admissions, 37.84%, n = 2 [67,68]	58.3%, n = 1 [76]	0.4 admissions, n = 1 [54]
Hospital admissions (number of admissions per year, % of patients)	2.6 admissions, 91.3%, n = 8 [33–35,41,48,51,65,83]	0.1 admissions, n = 1 [42]	0.6 admissions, n = 1 [41]	-	0.3 admissions, 69.5%, n = 2 [36,49]	76.8%, n = 1 [76]	3.7 admissions, n = 1 [54]
LOS in the hospital (number of days per year)	16.1 days, n = 7 [33,34,41,46,48,51,83]	-	5.8 days, n = 1 [41]	-	8.3 days, n = 3 [49,52,67]	-	-
Outpatient visits (number of visits, percentage of patients)	65.8 visits, n = 4 [41,51,56,83]	-	170.3 visits, n = 1 [41]	-	-	7.5 visits, 96.8%, n = 2 [76,81]	-
Physiotherapy (number of visits, percentage of patients)	14.7 visits, 64.6% n = 2 [37,87]	15.4 visits, 67.1%, n = 1 [37]	14.7 visits, 71.9%, n = 1 [37]	9.9 visits, 59.5%, n = 1 [37]	-	82.5%, n = 1 [47]	-
Speech therapy (number of visits, percentage of patients)	4.5 visits, 41.2%, n = 1 [37]	6.4 visits, 31.9%, n = 1 [37]	5.1 visits, 26.2%, n = 1 [37]	4.1 visits, 15.9%, n = 1 [37]	-	-	-
Medical care (medical consultation, assessing, clinical evaluation) (number of visits, percentage of patients)	2.5 visits, 19.6%, n = 1 [37]	2.3 visits, 18.3%, n = 1 [37]	2.3 visits, 15.4%, n = 1 [37]	2.3 visits, 14.6%, n = 1 [37]	-	-	-
Wheelchair usage (percentage of patients)	13.5%, n = 2 [37,87]	24.2%, n = 1 [37]	32.5%, n = 1 [37]	24.9%, n = 1 [37]	-	-	-
Orthosis usage (percentage of patients)	18.1%, n = 2 [37,59]	31.5%, n = 1 [37]	40.8%, n = 1 [37]	14.1%, n = 1 [37]	-	-	-
Home service/nurse (number of visits, percentage of patients)	38.3 visits, 13%, n = 2 [41,83]	-	27.0 visits, n = 1 [41]	-	-	-	-
Drug administration (nusinersen) (percentage of patients)	2.92 administrations, 9.28%, n = 1 [37]	3.55 administrations, 8.56%, n = 1 [37]	2.89 administrations, 5.38%, n = 1 [37]	2.93 administrations, 0.71%, n = 1 [37]	-	-	-
Laboratory tests (number of tests per year, percentage of patients)	0.73 tests, 0.69%, n = 2 [37,87]	1.73 tests, 0.67%, n = 1 [37]	1.68 tests, 0.93%, n = 1 [37]	1.1 tests, 0.25%, n = 1 [37]	-	-	91.07 tests, n = 1 [53]

Eight studies reported that type 1 SMA patients experience an average of 2.6 hospital admissions per year, with 91.3% of these patients requiring hospitalization due to severe progression of the disease. However, due to less severe disease progression and fewer consequences, SMA types 2 and 3 patients experience fewer hospitalizations, with an average of 0.13 and 0.56 admissions per year, respectively. Compared to other subtypes, the hospital length of stay (LOS) for type 1 SMA patients was 16.1 days annually (n = 7 studies), while unspecified types have an average LOS of 8.31 days, indicating substantial healthcare demands. Type 3 patients have a shorter average LOS of 5.8 days.

Interestingly, type 3 SMA has a higher rate of outpatient visits (170.3 per year) within a single study. Type 1 SMA patients, on the other hand, receive 65.76 visits annually on average, as reported by four studies. Although the frequency and percentage of patients vary, all forms of SMA show a considerable level of participation in speech and physical therapies. The average number of physiotherapy sessions for patients with type 1 SMA is 14.65 (64.61%), and the average number of speech therapy visits is 4.45 (41.23%), indicating the continuous requirement for supporting therapies to preserve function and communication skills.

Type 3 SMA patients have high percentages of wheelchair and orthosis usage (32.47% and 40.82%, respectively), compared to none for other SMA types. On average, type 1 SMA patients receive 38.26 home service or nurse visits annually. Because of routine monitoring and outpatient care, laboratory testing is more prevalent in type 2 and type 3 SMA than in type 1. Other studies reporting findings on types of SMA in an aggregated manner are included in Supplemental material 7 for a more thorough understanding of the HCRU across all SMA types.

Productivity loss

Only 10 studies included data about productivity loss due to SMA. The data were quantified by time lost in hours due to absenteeism and/or presenteeism, unspecified working time lost, or time lost by caregivers in daily care for SMA patients.

Six studies reported time lost by caregivers for caring for SMA patients [35,40,50,63,66,67]. Two studies reported unspecified working time lost by caregivers to care for SMA patients, and two studies reported time lost due to absenteeism. There were no studies reporting working time lost due to presenteeism. Since the data were limited, we aggregated all productivity loss values and calculated the average time lost per patient or per caregiver, in hours as shown in Table 4. SMA type 1 patient average caregiving time per year was 2,947 hours, whereas SMA type 3 average caregiving time was 537 hours.

**Table 4:** Average annual time lost in hours by each SMA type The table shows the number of hours lost annually by patients or caregivers due to SMA differentiated by SMA subtype and the sources used for these average value calculations. n: number of studies; SMA: spinal muscular atrophy

Time lost	Type 1 only		Type 2 only		Type 3 only		Unspecified		Types 1, 2, & 3	
	Time lost (hours)	Studies reporting time lost	Time lost (hours)	Studies reporting time lost	Time lost (hours)	Studies reporting time lost	Time lost (hours)	Studies reporting time lost	Time lost (hours)	Studies reporting time lost
Caregiver	2,947	n = 3 [69,80,87]	1,692	n = 3 [69,80,87]	537	n = 2 [69,80]	2,607	n = 1 [58]	2,540	n = 5 [38,74,76,80,81]
Patient	0	n = 1 [69]	169	n = 1 [69]	354	n = 1 [69]	-	-	83	n = 2 [76,81]

Discussion

SMA is a rare genetic disease that causes a significant economic burden to patients, caregivers, and the healthcare system. The impact varies between subtypes, as patients with types 1 and 2 usually have more severe morbidities and consume more resources compared to later-onset forms. Based on our review of 55 studies, critical gaps were identified in the literature related to productivity loss, particularly absenteeism and presenteeism.

Concerning costs, direct medical costs were the primary driver for cost among all SMA subtypes, with type 1 SMA having the highest cost at a weighted average of US$187,881, substantially exceeding type 2 (US$50,508) and type 3 (US$77,942). Dangouloff et al. found that advanced treatments and hospitalization were the primary contributors to type 1 SMA costs [19]. Similarly, Paracha et al. highlighted the disproportionate resource utilization for type 1 patients [16]. In addition to medical costs, type 1 SMA had the highest direct non-medical costs, including caregiver support and home modifications, averaging US$109,379, as noted by Landfeldt et al. [20]. Indirect costs, primarily from productivity losses due to caregiving demands, were also highest for type 1 patients at US$25,070, consistent with findings from Brandt et al. [18].

Furthermore, the weighted average of the total annual cost per patient was US$109,096 (range: US$3,752-US$593,517). According to Yang et al. [17], this variation is an indication of discrepancies in healthcare systems, treatment guidelines, and economic perspectives across countries. Cross-study comparisons become more challenging with the absence of defined cost-reporting approaches, underscoring the necessity of standardized frameworks for better economic evaluations. Policymakers will be able to create focused interventions for different SMA subtypes and more efficiently allocate resources if these gaps are filled [16].

Regarding HCRU across various types of SMA, nearly half of the studies indicate that type 1 patients require frequent hospital admissions and extended LOS, due to the relatively higher disease severity and intensive care unit admissions [33,41,65]. The disease burden entails unnegotiable medical services required by these patients, due to muscle weakness/failure. These services include respiratory and nutritional supports, provided by a cough-assist device and gastrostomy, respectively, as shown in Supplemental material 9. This burden further extends to other required therapies for the proper functionality of the patient, such as physiotherapy, occupational therapy, and speech therapy [37,87]. Logically, SMA types 2 and 3, due to less disease severity, have a lower hospitalization rate yet require higher outpatient visits. This emphasizes the urge for effective outpatient service management [41,42].

Additionally, Barbour et al. found that type 3 SMA is associated with a higher likelihood of physical disability, reflected in the increased use of mobility aids such as wheelchairs and orthoses. The study also highlighted the frequent pharmacological interventions among type 2 SMA patients, particularly the administration of nusinersen, underscoring the consistent demand for specific treatments [37]. Although numerous studies focused on the severe clinical consequences of type 1 SMA, data underline the disproportionate use of healthcare resources for this disorder. The results highlight the need for a resource-intensive approach to manage type 1 SMA, while outpatient and supportive care strategies may be more appropriate for types 2 and 3 [88].

Only six studies highlighted the productivity loss, an important yet underreported aspect of the financial burden of SMA. Based on these studies, type 1 patients require considerable care, consuming an average of 2,947 hours annually by their caregivers. A significant gap is the lack of information on presenteeism and the associated costs, considering this as an undetected burden on families and society [38,74,76,80,81,87].

Our SLR provides a comprehensive analysis of costs across SMA subtypes, excluding disease-modifying treatment (DMT) costs, building on and expanding seven prior systematic reviews. Unlike Yang et al., which focused solely on DMTs and health-related quality of life, we offer a broader economic perspective [17]. While Landfeldt et al. analyzed cost differences across countries and later addressed caregiver burden in 2023, we conducted a combined analysis of direct medical and non-medical costs, in addition to indirect costs [13,20].

Additionally, our review quantified productivity losses to address the financial and emotional implications, complementing Brandt et al.’s exploration of caregivers' psychological effects [18]. According to Dangouloff et al., the annual direct medical costs for SMA type 1 range between US$50,000 and US$160,000, whereas our weighted average of US$187,881 reflects an updated methodology and expanded dataset [19]. Finally, while Paracha et al. analyzed HCRU, we extended this with a larger dataset and detailed cost breakdowns by subtype [16].

Strengths

In order to give insights that are essential for focused financial and operational planning and resource allocation, our evaluation provides a thorough and reliable analysis of the economic cost of SMA by separately breaking down data by subtypes (types 1, 2, and 3). With the use of gray literature and reference snowballing, we were able to ensure thorough data capture and reduce gaps in our extended search across databases such as Embase and Medline. A thorough classification of the cost data into direct medical, direct non-medical, and indirect expenditures enables a granular analysis of HCRU and productivity losses across SMA subtypes.

Limitations

Our study acknowledges a number of limitations in synthesizing diverse data sources, which may impact the generalizability of the findings. Although numerous studies have been published on SMA, fewer studies were considered in each analysis due to the variability of the findings. This is because analyses were carried out independently for every subtype. To properly estimate the disease's economic impact, subtype-specific research is still needed, as each SMA subtype corresponds to different severity and prognostic factors and, thereby, different cost estimates.

The findings possess higher credibility as data were retrieved using weighted averages from various studies. However, only a few studies have reported the loss of productivity incurred by patients or caregivers, underscoring the need for further research in this area. Furthermore, information for certain outcomes, like resource utilization, was obtained from one investigation. Hence, values obtained for radiological testing, pediatric dietitian visits, and psychologist visits may be less reliable.

## Conclusions

The substantial economic impact of SMA, which varies by subtype and geographic location, is highlighted in this SLR. The findings demonstrate the necessity to standardize economic reporting to enhance the value and comparability of studies. Bridging these gaps might allow policy- and decision-makers and authorities to better aid SMA in managing their resources.

Future studies should standardize cost categories and terms to improve comparability and comprehension of the economic impact of SMA. Longitudinal studies and modeling are still required to evaluate the long-term economic impact on families and healthcare systems. Assess indirect costs to provide a more comprehensive global perspective, particularly in neglected areas.

## Figures and Tables

**Figure 3:**
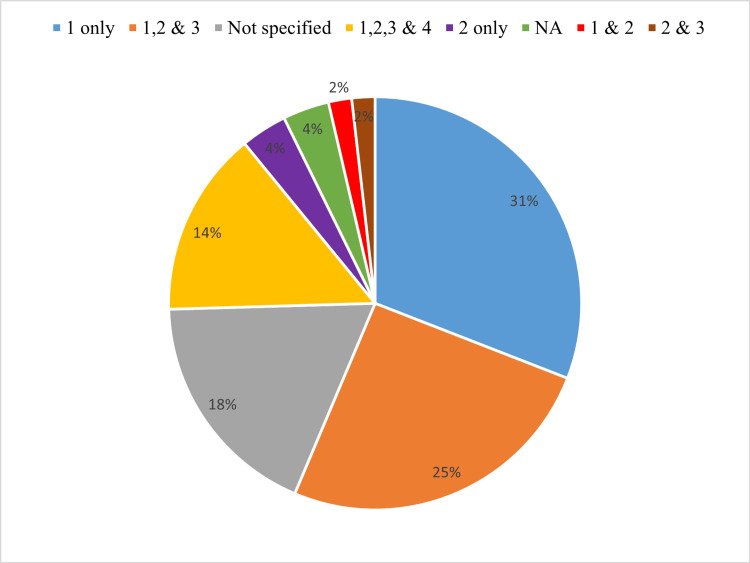
SMA subtypes of included studies (N = 55 studies) The figure shows the distribution of studies reporting economic data for SMA by subtype. Most studies reported data for SMA type 1 only (31%), followed by types 1, 2, and 3 combined (25%). Some studies (18%) did not specify the subtype, while 14% included types 1, 2, 3, and 4. Additionally, smaller proportions were reported on other less common categorizations. SMA: spinal muscular atrophy

## Appendices

Supplemental material 1

**Table 5:** Search term domains and their synonyms used for the search process SMA: spinal muscular atrophy

Number	Domain 1	Domain 2	Domain 3	Domain 4
Domain	SMA	Cost of illness	Productivity lost	Healthcare resource utilization
Synonyms	SMA, Spinal muscular atrophy, Werdnig-Hoffmann, Kugelberg-Welander	Cost, Costs, Costing, Economic burden, Price, Prices, Expenditure, Expenditures, Financial, Financials, Monetary, Expense, Expenses	Productivity lost, Presenteeism, Absenteeism, Lost productivity	Utilization, Utilisation, Hospitalization, Hospitalizations, Hospitalisation, Hospitalisations, Visit, Visits, Admissions, Admission

Supplemental material 2 

**Table 6:** Search strategy and results

Source	Search term	# of hits
PubMed	(("SMA"[Title/Abstract] OR "spinal muscular atrophy"[Title/Abstract] OR "Werdnig-Hoffmann"[Title/Abstract] OR "Kugelberg-Welander"[Title/Abstract]) AND ("Cost"[Title/Abstract] OR "Costs"[Title/Abstract] OR "Costing"[Title/Abstract] OR "Economic burden"[Title/Abstract] OR "price"[Title/Abstract] OR "prices"[Title/Abstract] OR "expenditure"[Title/Abstract] OR "expenditures"[Title/Abstract] OR "Monetary"[Title/Abstract] OR "Financial"[Title/Abstract] OR "Financials"[Title/Abstract] OR "Expense"[Title/Abstract] OR "Expenses"[Title/Abstract] OR "Productivity lost"[Title/Abstract] OR "Lost productivity"[Title/Abstract] OR "Presenteeism"[Title/Abstract] OR "Absenteeism"[Title/Abstract] OR "Utilization"[Title/Abstract] OR "Utilisation"[Title/Abstract] OR "Hospitalization"[Title/Abstract] OR "Hospitalizations"[Title/Abstract] OR "Hospitalisation"[Title/Abstract] OR "visit"[Title/Abstract] OR "visits"[Title/Abstract] OR "Admissions"[Title/Abstract] OR "Admission"[Title/Abstract] OR "Hospitalisations"[Title/Abstract])) AND (2010/1/1:2022/3/17[pdat])	595 hits
Scopus	TITLE-ABS-KEY ((“SMA" OR "spinal muscular atrophy" OR "Werdnig-Hoffmann" OR "Kugelberg Welander" OR "" ) AND ( "" OR "Cost" OR “Costs” OR “Costing” OR “Economic burden" OR "price” OR “prices" OR “expenditure" OR "expenditures" OR "Monetary" OR "Financial" OR "Financials" OR "Expense" OR "Expenses" OR "Productivity lost" OR "Lost productivity" OR "Presenteeism" OR "Absenteeism" OR "Utilization" OR "Utilisation" OR "Hospitalization" OR "Hospitalizations" OR "Hospitalisation" OR "visit" OR "visits" OR "Admissions" OR "Admission" OR "Hospitalisations" ) ) AND ( LIMIT-TO ( SUBJAREA , "MEDI" ) ) AND ( LIMIT-TO ( PUBYEAR , 2022 ) OR LIMIT-TO ( PUBYEAR , 2021 ) OR LIMIT-TO ( PUBYEAR , 2020 ) OR LIMIT-TO ( PUBYEAR , 2019 ) OR LIMIT-TO ( PUBYEAR , 2018 ) OR LIMIT-TO ( PUBYEAR , 2017 ) OR LIMIT-TO ( PUBYEAR , 2016 ) OR LIMIT-TO ( PUBYEAR , 2015 ) OR LIMIT-TO ( PUBYEAR , 2014 ) OR LIMIT-TO ( PUBYEAR , 2013 ) OR LIMIT-TO ( PUBYEAR , 2012 ) OR LIMIT-TO ( PUBYEAR , 2011 ) OR LIMIT-TO ( PUBYEAR , 2010 ) )	847 hits
Gray literature (Google search)	Spinal muscular atrophy burden	The first 100 hits were screened

Supplemental material 3 

**Table 7:** Data extraction sheet domains DMT: disease-modifying treatments; SMA: spinal muscular atrophy

Data domain	Data collected
General information	Country, Starting and end period, Number of patients or caregivers, Type of SMA (0-4 classification), Age
Cost	Cost types, In case of indirect cost is it for patient/caregiver?, Cost value, Currency, Period/Period unit, Perspective, Were costs of DMTs included?, Types of DMTs included, Other treatment if any, Is it the actual or estimated cost?, Year of the reported cost
Healthcare resource utilization	Healthcare resource type, Details about healthcare resources, Utilization value, Period, DMTs included?, Types of DMTs
Productivity loss	Productivity loss type, Details about productivity loss, For patient or caregiver, Productivity loss value, Period, DMTs included?, Types of DMTs included, Other treatment if any

Supplemental material 4 

Supplemental material 5 

**Table 8:** Summary of included studies with quality assessment results

#	Author (year)	Study type	Quality of study
1	Ali et al., 2019 [33]	Cohort	Unsatisfactory
2	Al-Zaidy et al., 2019 [34]	Cohort	Good
3	Arjunji et al., 2019 [35]	Cohort	Unsatisfactory
4	Armstrong et al., 2016 [36]	Cohort	Good
5	Barbour et al., 2021 [37]	Cohort	Good
6	López-Bastida et al., 2017 [38]	Cross-sectional	Good
7	Lopez Bastida et al., 2019 [39]	Cross-sectional	Very good
8	Beauchamp et al., 2019 [40]	Economic	97
9	Belter et al., 2020 [41]	Cross-sectional	Good
10	Bielsky et al., 2018 [42]	Cohort	Good
11	Bladen et al., 2014 [43]	Cross-sectional	Very good
12	Broekhoff et al., 2021 [44]	Economic	94
13	CADTH, 2019 [45]	Economic	86
14	Cardenas et al., 2019 [46]	Cohort	Good
15	Chambers et al., 2020 [47]	Cross-sectional	Good
16	Chen et al., 2021 A [48]	Cohort	Good
17	Chen et al., 2021 B [49]	Cross-sectional	Very good
18	Cremers et al., 2019 [50]	Cross-sectional	Very good
19	Dabbous et al., 2018 [51]	Cohort	Good
20	Darbà and Marsà, 2019 [52]	Cohort	Good
21	Darbà, 2020 A [53]	Cohort	Good
22	Darbà et al., 2020 B [54]	Cohort	Good
23	Dean et al., 2021 [55]	Economic	87
24	Droege et al., 2020 A [56]	Cohort	Good
25	Droege et al., 2020 B [57]	Cohort	Good
26	Farrar et al., 2018 [58]	Cohort	Unsatisfactory
27	Fox, 2020 [59]	Cohort	Good
28	García-Salido et al., 2015 [60]	Cohort	Unsatisfactory
29	Gauthier-Loiselle et al., 2021 [61]	Cohort	Good
30	Gonçalves et al., 2021 [62]	Cross-sectional	Very good
31	Han et al., 2015 [63]	Cohort	Good
32	Hully et al., 2020 [64]	Cohort	Good
33	ICER, 2019 [65]	Economic	100
34	Jalali et al.,2020 [66]	Economic	91
35	Johnson et al., 2021 [67]	Cohort	Good
36	Kao et al., 2019 [68]	Cohort	Good
37	Klug et al., 2016 [69]	Cross-sectional	Good
38	Lee et al.,2019 [70]	Cohort	Good
39	Lemoine et al., 2012 [71]	Cohort	Good
40	The Lewin Group, 2012 [72]	Cross-sectional	Good
41	Lomba et al., 2021 [73]	Cohort	Good
42	Peña-Longobardo et al., 2020 [74]	Cross-sectional	Good
43	Malone et al., 2019 [75]	Economic	100
44	McMillan et al., 2021 [76]	Cross-sectional	Good
45	Moran et al., 2015 [77]	Cross-sectional	Unsatisfactory
46	NICE, 2019 [78]	Economic	94
47	Ottonello et al., 2011 [79]	Cohort	Good
48	Aranda-Reneo et al., 2020 [80]	Cross-sectional	Good
49	Rowell et al., 2020 [81]	Cross-sectional	Unsatisfactory
50	Shih et al., 2021 [82]	Economic	94
51	Tan et al., 2019 [83]	Cohort	Good
52	Tassie et al., 2013 [84]	Cohort	Good
53	Tetafort et al., 2017 [85]	Cohort	Good
54	Thokala et al., 2020 [86]	Economic	94
55	Zuluaga‑Sanchez et al., 2019 [87]	Economic	88

Supplemental material 6 

**Table 9:** Countries involved in the included studies

Country	Number of studies involved
United States of America	21
United Kingdom	8
Spain	7
Australia	6
France	6
Germany	5
Canada	4
Netherlands	3

Supplemental material 7 

**Table 10:** Average weighted annual cost per SMA patient (all SMA types) in US$ *Data was based on estimates from a small sample size as reported by the study. US$: United States dollar; SMA: spinal muscular atrophy

Cost type	Weighted average	Studies reporting the values	Minimum cost	Maximum cost
Total cost (US$)	109,096	n = 7 [40,47,69,70,72,82,87]	3,752 [47]	593,517 [40]
Total direct cost (US$)	58,412	n = 5 [38,47,69,74,87]	2,103 [47]	180,052 [87]
Direct medical cost (US$)	59,570	n = 19 [36,38,39,44,45,49,51,54,56,59,61,67,69,71,72,78,82,83,87]	0* [72]	334,715 [51]
Direct non-medical cost (US$)	39,910	n = 5 [38,69,72,74,87]	16,967 [72]	141,893 [87]
Indirect cost (US$)	18,025	n = 4 [47,72,87]	1,649 [47]	58,796 [87]

Supplemental material 8 

**Table 11:** Average of different cost types of each SMA subtype reported by included studies US$: United States dollar; SMA: spinal muscular atrophy

Study name	Total cost (US$)	Total direct cost (US$)	Direct medical cost (US$)	Direct non-medical cost (US$)	Indirect cost (US$)
Type 1 only					
Broekhoff et al., 2021 [44]	-	-	10,454	-	-
CADTH, 2019 [45]	-	-	10,754	-	-
Chambers et al., 2020 [47]	59,876	48,363	-	-	11,513
Dabbous et al., 2018 [51]	-	-	334,715	-	-
Fox, 2020 [59]	-	-	235,198	-	-
Droege et al., 2020 A [56]	-	-	95,460	-	-
Gauthier-Loiselle et al., 2021 [61]	-	-	79,079	-	-
Klug et al.,2016 [69]	129,128	119,736	64,523	55,213	13,277
Lemoine et al., 2012 [71]	-	-	69,946	-	-
NICE, 2019 [78]	-	-	54,426	-	-
Tan et al., 2019 [83]	-	-	334,360	-	-
Zuluaga‑Sanchez et al., 2019 [87]	174,633	173,548	31,670	141,878	58,796
Type 2 only					
Lopez Bastida et al., 2019 [39]	-	43,571	13,394	30,176	-
CADTH, 2019 [45]	-	-	11,217	-	-
Chambers et al., 2020 [47]	6,061	3,124	-	-	2,937
Gauthier-Loiselle et al., 2021 [61]	-	-	100,450	-	-
Klug et al., 2016 [69]	108,437	88,797	18,387	70,410	20,987
Type 3 only					
CADTH, 2019 [45]	-	-	107,965	-	-
Chambers et al., 2020 [47]	9,919	5,453	-	-	4,466
Gauthier-Loiselle et al., 2021 [61]	-	-	89,836	-	-
Klug et al., 2016 [69]	63,018	43,327	10,963	32,366	16,610
Types 1, 2, & 3					
Lopez Bastida et al., 2019 [39]	-	39,004	12,587	26,417	-
Chambers et al., 2020 [47]	3,752	2,103	-	-	1,649
Klug et al., 2016 [69]	84,766	65,742	17,230	48,510	19,753
The Lewin Group, 2012 [72]	219,140	-	101,153	61,321	21,078
Peña-Longobardo et al., 2020 [74]	-	55,297	9,247	46,050	-
Shih et al., 2021 [82]	201,292	-	26	-	-
Type not specified					
Armstrong et al., 2016 [36]	-	-	158,833	-	-
Beauchamp et al., 2019 [40]	464,091	-	-	-	-
Chen et al., 2021 B [49]	-	-	5,375	-	-
Johnson et al., 2021 [67]	-	-	1,662	-	-
Lee et al., 2019 [70]	63,656	-	-	-	-

Supplemental material 9 

**Table 12:** Average annual healthcare resource utilization (HCRU) per SMA type BiPAP: bilevel positive airway pressure; CPAP: continuous positive airway pressure; ICU: intensive care unit; n: number of studies; PICU: pediatric intensive care unit

HCRU	Type 1 only	Types 1, 2, & 3	Types 1, 2, 3, & 4	Type 2 only	Type 3 only	Type 4 only	Not specified
Diagnostics							
Laboratory tests (number of tests per year, percentage of patients)	0.73 tests, 0.69%, n = 2 [37,87]	-	91.07 tests, n = 1 [37]	1.73 tests, 0.67%, n = 1 [37]	1.68 tests, 0.93%, n = 1 [37]	1.1 tests, 0.25%, n = 1 [37]	-
Radiologic testing (percentage of patients)	95.7%, n = 1 [83]	-	-	-	-	-	-
Emergency room visits							
Emergency room admissions (number of admissions per year, percentage of patients)	1.27 admissions, 78.45%, n = 5 [33,48,71,76,83]	58.3%, n = 1 [76]	0.38 admissions, n = 1 [53]	51.2%, n = 1 [76]	36.9%, n = 1 [76]	-	0.3 admissions, 37.84%, n = 2 [67,68]
Hospitalization							
Hospital admissions (number of admissions per year, percentage of patients)	2.6 admissions, 91.3%, n = 8 [33–35,41,48,51,56,83]	76.8%, n = 1 [76]	3.7 admissions n = 1 [53]	0.13 admissions, n = 1 [42]	0.56 admissions, n = 1 [41]	-	0.3 admissions, 69.5%, n = 2 [36,49]
Length of stay in the hospital (number of days per year)	16.1 days, n = 7 [33,34,41,46,48,51,83]	-	-	-	5.8 days, n = 1 [41]	-	8.31 days, n = 3 [49,54,67]
Length of stay in ICU/PICU (number of days per year)	7.74 days, n = 4 [33,48,84,87]	-	-	-	-	-	2.73 days, n = 1 [67]
ICU/PICU admissions (number of admissions per year, percentage of patients)	0.62 admissions, 48.6%, n = 2 [48,84]	-	-	-	-	-	28.99%, n = 1 [67]
Respiratory hospitalization (number of admissions per year, percentage of patients)	0.15 admissions, 10.84 days, 67.15%, n = 5 [34,56,57,71,79]	-	-	-	-	-	-
Cough-assist device (percentage of patients)	46.94%, n = 5 [33,48,71,83,87]	-	-	-	-	-	-
Gastrostomy (number of admissions per year, percentage of patients)	0.18 admissions, 0.45 time, 64.67%, n = 4 [48,71,83,87]	-	-	-	-	-	-
Orthopedic surgery (percentage of patients)	32.01%, n = 2 [59,83]	-	-	-	-	-	-
Outpatient							
Outpatient visits (number of visits, percentage of patients)	65.76 visits, n = 4 [41,51,56,83]	7.5 visits, 96.8%, n = 2 [76,81]	-	-	170.3 visits, n = 1 [41]	-	-
Outpatient/non-specialist (number of visits, percentage of patients)	17.26 visits, 8.7%, n = 2 [41,83]	-	-	-	14.76 visits, n = 1 [41]	-	0.57 visits, n = 1 [49]
Specialist visits (number of visits)		-	-	-	-	-	0.55 visits, n = 1 [49]
Pediatric dietitian (number of visits)	12 visits, n = 1 [87]	-	-	-	-	-	-
Pulmonologist (number of visits, percentage of patients)	2.5 visits, 56.91%, n = 3 [83,84,87]	-	-	-	-	-	-
Psychologist (number of visits, percentage of patients)	2.5 visits, n = 1 [87]	7.5%, n = 1 [47]	-	-	-	-	-
Neurologist (number of visits, percentage of patients)	4 visits, 82.76%, n = 3 [83,84,87]	-	-	-	-	-	-
Respiratory support							
Respiratory support (percentage of patients)	55.28%, n = 1 [59]	-	-	-	-	-	-
Non-invasive ventilation (percentage of patients)	57.91%, n = 4 [33,34,48,84]	-	-	-	-	-	14.6%, n = 1 [36]
Invasive ventilation (percentage of patients)	17.89%, n = 3 [33,71,84]	8.15%, n = 1 [72]	-	-	-	-	-
BiPAP ventilation (device usage annually, percentage of patients)	0.5 times, 46.94%, n = 2 [71,87]	-	-	-	-	-	-
CPAP ventilation (device usage annually, percentage of patients)	0.5 times, n = 1 [87]	-	-	-	-	-	-
Rehabilitation							
Occupational therapist (number of visits, percentage of patients)	4 visits, n = 1 [87]	75%, n = 1 [47]	-	-	-	-	-
Physiotherapy (number of visits, percentage of patients)	14.65 visits, 64.61%, n = 2 [37,87]	82.5%, n = 1 [47]	-	15.43 visits, 67.11%, n = 1 [37]	14.66 visits, 71.99%, n = 1 [37]	9.91 visits, 59.54%, n = 1 [37]	-
Speech therapy (number of visits, percentage of patients)	4.45 visits, 41.23%, n = 1 [37]	-	-	6.43 visits, 31.88%, n = 1 [37]	5.1 visits, 26.16%, n = 1 [37]	4.12 visits, 15.85%, n = 1 [37]	-
Counselor visit (number of visits, percentage of patients)	4 visits, n = 1 [87]	36.5%, n = 1 [76]	-	-	-	-	-
Other healthcare resource utilization							
Cough-assist device (percentage of patients)	72.95%, n = 5 [33,48,71,83,87]	-	-	-	-	-	-
Feeding support (percentage of patients)	17.87%, n = 3 [59,83,84]	-	-	-	-	-	-
Medical care (medical consultation, assessing, clinical evaluation) (number of visits, percentage of patients)	2.47 visits, 19.59%, n = 1 [37]	-	-	2.25 visits, 18.29%, n = 1 [37]	2.33 visits, 15.4%, n = 1 [37]	2.25 visits, 14.62%, n = 1 [37]	-
Orthopedic surgery (percentage of patients)	17.4%, n = 2 [59,83]	-	-	-	-	-	-
Scoliosis surgery (number of procedures, percentage of patients)	0 procedures, 4.3%, n = 2 [83,87]	-	-	-	-	-	-
Suction (percentage of patients)	69.66%, n = 4 [33,71,83,87]	-	-	-	-	-	-
Wheelchair usage (percentage of patients)	13.5%, n = 2 [37,87]	-	-	0.95 use, 24.16%, n = 1 [37]	1.08 use, 32.47%, n = 1 [37]	1.09 use, 24.88%, n = 1 [37]	-
Orthosis usage (percentage of patients)	18.06%, n = 2 [37,59]	-	-	31.54%, n = 1 [37]	40.82%, n = 1 [37]	14.06%, n = 1 [37]	-
Home service/nurse (number of visits, percentage of patients)	38.26 visits, 13%, n = 2 [41,83]	-	-	-	27.03 visits, n = 1 [41]	-	-
Drug administration (nusinersen) (percentage of patients)	2.92 administrations, 9.28%, n = 1 [37]	-	-	3.55 administrations, 8.56%, n = 1 [37]	2.89 administrations, 5.38%, n = 1 [37]	2.93 administrations, 0.71%, n = 1 [37]	-
